# Factors associated with health-related quality of life in women with paid work at breast cancer diagnosis: a German repeated cross-sectional study over the first five years after primary surgery

**DOI:** 10.1186/s12885-025-13491-8

**Published:** 2025-01-17

**Authors:** Batoul Safieddine, Siegfried Geyer, Stefanie Sperlich, Johannes Beller, Dorothee Noeres

**Affiliations:** Medical Sociology Unit, Hannover Medical School, Hannover, Germany

**Keywords:** Breast cancer, Quality of life, Short-form health survey, Return to work, Socioeconomic factors

## Abstract

**Background:**

Evidence suggests a deterioration of health-related quality of life (HRQL) after breast cancer diagnosis and therapy. This study examines sociodemographic and health-related factors that could be associated with the HRQL of working women with breast cancer during the first five years after primary surgery. Second, it explores potential vulnerable groups with respect to HRQL using decision tree analyses.

**Methods:**

Women diagnosed with breast cancer who had paid work at diagnosis were recruited at 11 breast cancer centers in the Hannover region, Germany, after primary surgery. Assessments took place four times. 455 patients completed mailed questionnaires at 3 weeks after primary surgery. Women were followed up at 6 months, 1 year and on average 5 years after primary surgery. The physical and mental wellbeing dimensions of HRQL were examined through the Short-Form health survey-12. Potential associations between HRQL and health and sociodemographic factors were examined using multiple linear regression. Classification tree analyses were applied to define specific vulnerable groups.

**Results:**

Mastectomy (ß=-2.49; CI:-4.67, -0.30) and chemotherapy (ß=-4.25; CI:-7.04, -1.46) as health related factors were significantly associated with poorer physical wellbeing at 3 weeks and 6 months after primary surgery, respectively. Returning to work (RTW) after having been on sick leave was strongly associated with better HRQL as illustrated by higher sum scores for physical (at 3 weeks: ß=6.21; CI:3.36, 9.05; at 6 months: ß=5.40; CI:3.01, 1.80; at 1 year: ß=8.40; CI:5.31, 11.49) and mental wellbeing (at 6 months: ß=6.03; CI:33.25, 8.81; at 1 year: ß=7.71; CI:4.85, 10.58) until 1 year after primary surgery. However, its significant effect was no more apparent at 5 years after primary surgery. At that stage, income was mostly associated with physical (ß=0.002; CI:0.0002, 0.003) and mental wellbeing (ß=0.002; CI:0.0005, 0.003) with higher summary scores for higher income especially in women aged ≤ 61 years. In addition, living with a partner appeared to be an important positively associated factor with better mental wellbeing in women with breast cancer (at 6 months: ß=3.68; CI: 0.72, 6.63; at 5 years: ß=2.85; CI:0.39, 5.32) and the first splitting node that defined vulnerability at 5 years.

**Conclusions:**

HRQL in breast cancer appears to be a multidimensional phenomenon associated with disease, treatment and social factors. A special focus should be drawn to women with lower income and those not living with a partner when planning rehabilitation programs and strategies that aim to improve the long term HRQL in breast cancer. As RTW appeared to be positively associated with HRQL, future research should examine potential causal relationships between RTW and HRQL in breast cancer in order to provide evidence needed to plan prevention strategies that aim to improve HRQL after breast cancer.

**Supplementary Information:**

The online version contains supplementary material available at 10.1186/s12885-025-13491-8.

## Background

Breast cancer is the most common type of cancer and the leading cause of cancer-related deaths among women globally [1, 2]. In Germany, age-standardized breast cancer incident rates were reported to be 112.7 per 100,000 in 2020, which makes it by far the most incident type of cancer among women. Nevertheless, the five-year survival rate has been reported to be 88% in Germany [3]. Given the relatively early onset [4] and high survival rate [5, 6] of breast cancer, this disease is associated with a high individual and public health burden due to the relatively long duration of potential physical and social challenges and disability. Besides being the leading cause of disability adjusted life years among women [7], the economic burden of breast cancer is pronounced, especially due to loss of productivity and a decreased rate of return to work (RTW) [8].

The burden of breast cancer is highly attributable to the deteriorated health-related quality of life (HRQL) among survivors [7–9]. The treatment of breast cancer usually involves a combination of therapies which play an important role in improving prognosis among breast cancer patients, but often cause short- and long-term side effects that influence quality of life [10, 11]. Breast cancer has been shown to be associated with deterioration in several dimensions of quality of life such as physical [12], mental [13] and social wellbeing [14]. Moreover, breast cancer patients face serious personal and interpersonal challenges during the first year after diagnosis [15]. Shortly after diagnosis, women with breast cancer find themselves in a phase where they need to take serious decisions, deal with physical challenges caused by treatment, and mentally cope with the thought of having a disease that intimidates life and wellbeing [16, 17].

While studies point to a deteriorated HRQL in breast cancer survivors, evidence on the predictors of HRQL is controversial and varies between populations [18–21]. For instance, there has been a discrepancy in the results of studies on whether sociodemographic factors such as education or marital status [19, 21] play an important role in determining HRQL, or whether HRQL is determined by health-related variables such as disease stage or adjuvant therapies [19–21].

In employed women with breast cancer, factors associated with HRQL have been less commonly examined. Employed women might face additional challenges due to being on sick leave or quitting their jobs, which indicate a greater change in their daily routine and social life. The ability to cope with these challenges and the extent to which they influence HRQL depends on individual factors [22, 23]. Within this context, RTW could be an important potential determinant of HRQL among these women. Numerous studies on the impact of RTW on physical and mental health in women with breast cancer as well as generally in working-aged adults concluded a positive effect among a variety of populations, time periods and settings [24–26]. For example, in breast cancer survivors who were working before diagnosis, RTW has been shown to have a positive influence on quality of life as measured by a breast cancer specific instrument of HRQL [27]. Evidence also points towards a better global life satisfaction among breast cancer survivors who returned to work during the first year after diagnosis [28].

Nevertheless, understanding how different health-related and sociodemographic factors including RTW potentially intersect in affecting HRQL is essential to define specific vulnerable groups. In fact, understanding which factors are related to HRQL during the first years after diagnosis in employed women would provide an insight on individual variations that determine the HRQL, and whether the available evidence on the long term HRQL and its related factors applies during the acute critical phase after diagnosis. This would in turn strengthen the evidence required for planning interventions that aim to improve coping strategies and HRQL during the challenging period following diagnosis. Understanding whether early RTW is among the factors that affect HRQL would provide evidence for the design and budget planning of projects that aim to ease the RTW process such as occupational rehabilitation [29], gradual reintegration at work [30] and other potential support mechanisms [31] that would improve HRQL in this common disease.

In Germany, several studies examined quality of life in breast cancer, most of them considering a specific time point or focused on specific determinants [32–34]. Our study aims to examine sociodemographic and health factors that could be associated with HRQL during the first five years after primary surgery in order to define specific vulnerable groups.

Precisely, this study aims to:

1. Investigate sociodemographic and health-related factors associated with physical and mental wellbeing of working women with breast cancer at 3 weeks, 6 months, 1 year and 5 years after primary surgery.
2. Examine factors that characterize vulnerable groups with respect to physical and mental wellbeing at the above-mentioned time points.

## Methods

### Study design

The study population is based on a multicenter longitudinal study, primarily aimed at investigating RTW after breast cancer. Participants were recruited from 11 certified breast cancer centers in the Hannover region. Breast cancer patients were considered eligible if they were not older than 63 years, were working at the time of diagnosis, had undergone primary breast cancer surgery and did not exclusively have a ductal carcinoma in situ. All eligible patients were first contacted and introduced to the study through the breast cancer centers. After having given written informed consent, patients received the questionnaires by mail and sent them back in stamped addressed envelopes they also received. The first questionnaire was sent 3 weeks after the date of the primary surgical treatment. This was followed up by a second questionnaire at 6 months, a third questionnaire at 1 year after and a fourth questionnaires at, on average, 5 years after the date of primary surgery. In order to ensure high response and follow up rates, the guidelines developed by Dillman were followed [35]. These involved sending up to three reminders per wave in case the women did not send back their completed questionnaires. Medical data were extracted from medical reports. Recruitment and the first survey started in November 2016 and the follow-up ended in November 2019. Questionnaires used for the four time points after breast cancer primary surgery were designed by the research team for the purpose of the project. An English translated version of the items used for the analyses in this study is found in supplement file 1. The datasets used and analyzed in this study are available from the corresponding author on reasonable request.

The study was performed in accordance with the ethical standards as laid down in the 1964 Declaration of Helsinki and its later amendments or comparable ethical standards. The study was approved by the ethics committee of Hanover Medical School under the number 2973 − 2015.

Participants provided informed consent. In our data protection declaration, we pointed out that participation was voluntary and that there were no negative consequences for non-participation. Respondents received the declaration in the written invitation to the survey.

### Outcome

HRQL was examined with the first version of the Short Form health survey-12 (SF-12) [33]. The SF-12 licenses for the four questionnaires were obtained from the test center of Hogrefe Verlag GmbH & Co. (Testzentrale) under the customer number: 653,932. SF-12 is a commonly used instrument assessing HRQL in patients with chronic diseases. The validity and reliability of the SF-12 have been confirmed in more than 200 diseases, including breast cancer [36–38]. The two dimensions measured by the SF-12 are physical and mental wellbeing, each demonstrated by a score ranging between 0 and 100 with higher scores indicating better wellbeing. SF-12 scores were calculated according to German specific guidelines. German guidelines take into account cultural differences and norms of the German population and calculation of SF-12 is based on normative data from representative German samples [39]. While cancer specific instruments could provide more detailed information on the specific impact of breast cancer and its treatment, we used SF-12 to examine HRQL in order to enhance comparability of our results with other populations. Moreover, since the questionnaires in the study were relatively long, The SF-12 instrument that includes only 12 items was convenient due to better time efficiency and a reduced burden of participants, which might also affect response and follow up rates.

The study had eight outcomes: physical wellbeing and mental wellbeing at 3 weeks, 6 months, 1 year and 5 years after primary surgical treatment.

### Independent variables

#### Sociodemographic variables

Education was classified according to the highest achieved school diploma and is grouped into three categories: secondary general school (low) *(German designation: Hauptschulabschluss)*, intermediate secondary school (middle) *(German designation: Realschulabschluss)* and upper secondary school (high) *(German designation: Abitur)*. Occupational position was classified according to the classification used by the German Socioeconomic Panel [40]. It was divided into five groups according to the degree of autonomy. Category “1” denotes jobs with the lowest autonomy, such as unskilled manuals, and the level of autonomy increases with ascending categories with category “5” denoting jobs with the highest autonomy, such as senior employees self-employed workers. In the regression analyses, occupational position was grouped into three levels as follows: low autonomy (1&2), middle autonomy (3) & high autonomy (4&5). Income depicted the household income per person and was calculated by dividing total household income by the number of persons living in the household, weighted according to the age group of each. Weighting was done according to the organization for economic cooperation and development (OECD) modified equivalence scale that was adopted in 1990 [41]. Age was scaled in years, and living with partner, migratory background and RTW were dichotomous variables with the two answer categories: yes/no. All women who had returned to work by the time point (not only the new returnees at the corresponding time point) belonged to the “yes” category at the four time points respectively.

#### Health variables

The study considered disease stage which was defined according to the Union for International Cancer Control (UICC) into five main stages [42]. In the regression analyses, the five stages were summarized into three levels as follows: 1 (UICC 0 & UICC I), 2 (UICC II) and 3 (UICC III & UICC IV) (women with UICC 0 in the study had higher stages prior to neoadjuvant chemotherapy). In addition, surgical procedure indicating whether a breast-conserving treatment (BCT) or mastectomy was performed was considered. The study also considered the therapies: Chemotherapy, neoadjuvant chemotherapy, radiation therapy, anti-hormone therapy, rehabilitation, and being a member in a self-help group, each being coded as a dichotomous variable with the answer categories yes/no.

### Statistical analysis

Internal consistency of the SF-12 items was examined by Cronbach’s alpha, where α ≥ 0.70 was considered satisfactory [43]. Mean SF-12 scored for physical and mental wellbeing were calculated for each of the examined time points.

To examine factors associated with physical and mental wellbeing, linear regression analyses were applied. Sociodemographic factors (income, occupational autonomy at diagnosis, school education, living with a partner, migratory background and age), disease stage (UICC in three levels), type of surgery, therapies (chemotherapy, radiation therapy, antihormone therapy, neoadjuvant chemotherapy), rehabilitation and being a member in a self-help group were considered in this analyses. First univariate linear regression analyses were applied to examine the effect of each of the above-mentioned variables on physical and mental wellbeing. Second, in order to avoid statistical overfitting, only variables that were shown to have a significant effect at the univariate level were added to the multivariate regression analyses. Separate models were applied for physical and mental wellbeing and at each of the four time points, resulting in eight multivariate regression models.

The second line of analysis involved decision tree analyses examining intersectionality between potentially associated factors with physical and mental wellbeing, in order to identify factors that define specific vulnerable groups at different time points during the first 5 years after primary surgery. This analysis was essential since some examined factors could only be significant for specific subgroups and within explicit intersectionalities. Decision tree analyses were applied for physical and mental wellbeing at the four time points: 3 weeks, 6 months, 1 year and 5 years after primary surgery. The decision tree analysis identifies interactions within the independent variables, and displays a hierarchal illustration of their effects on the dependent variable. Factors explaining the maximum variance of the dependent variable will be illustrated as a first split in the decision tree, followed by potentially additional factors that interact and have significant effects on the outcome [44, 45]. The “Chi-squared Automatic Interaction Detection” (CHAID) set-up method was used. In each step, the CHAID method determines the independent variable that has the strongest correlation with the dependent variable. The categories of the individual independent variables are merged if they are not significantly different with regard to the dependent variable. Eight classification and regression trees were modeled for the eight outcomes: physical wellbeing and mental wellbeing at the four study time points. Physical and mental wellbeing were added to the models as metric variables. In all models, all sociodemographic and health related factors were added in the form described above as the independent variables. Significance levels were set at *p* < 0,05 for splitting nodes and for connecting categories in the independent variables. The minimum number of cases required was *n* = 100 for the parent node and *n* = 50 for the child nodes. The maximum number of levels for child nodes was set to three.

Statistical analyses were performed by means of STATA 15 and SPSS 25.

## Results

With a response rate of 80%, 455 women participated in the survey at 3 weeks after primary surgery. The follow-up rate was 95% at 6 months, 90% at 1 year, and 82% at 5 years after primary surgery. Mean age of the sample at baseline was 50.6 years, and most women had breast cancer at UICC-stages I and II. Most participants had a medium or high school education (42.2% and 41.1% respectively). Most women were in groups 2, 3 and 4 of job autonomy, with group 3 being dominant (41.1%). The household per person adjusted income at baseline was 1835 € and rose slightly over the study time points. Moreover, almost 20% of participants were born to at least one parent not born in Germany, which corresponds to the proportion of people with a migratory background in Germany [46]. Detailed sample characteristics are displayed in Table 1.

**Table 1:** Sample characteristics

*N*	
3 weeks	455
6 months	431
1 year	409
5 years	372
**Age at baseline (Sd)**	50.6 (7.4)
**Disease stage UICC** n(%)	
UICC 0	15 (3.3)
UICC I	219 (48.1)
UICC II	180 (39.6)
UICC III	34 (7.5)
UICC IV	6 (1.3)
**School education** n(%)	
Low	61 (13.4)
Middle	192 (42.2)
High	187 (3.3)
**Occupation** n(%)	
1 (*low autonomy)*	43 (9.5)
2	110 (24.2)
3	187 (41.1)
4	81 (17.8)
5 *(high autonomy)*	22 (4.8)
**Income** mean(SD)	
3 weeks	1835 (751)
6 months	1934 (798)
1 year	1886 (773)
5 years	2027 (799)
**Migratory Background** n(%) *baseline*	89 (19.6)
**RTW** n(%)	
3 weeks	45 (9.9)
6 months	234 (54.3)
1 year	312 (76.3)
5 years	215(57.8)

### HRQL

**Fig. 1:**
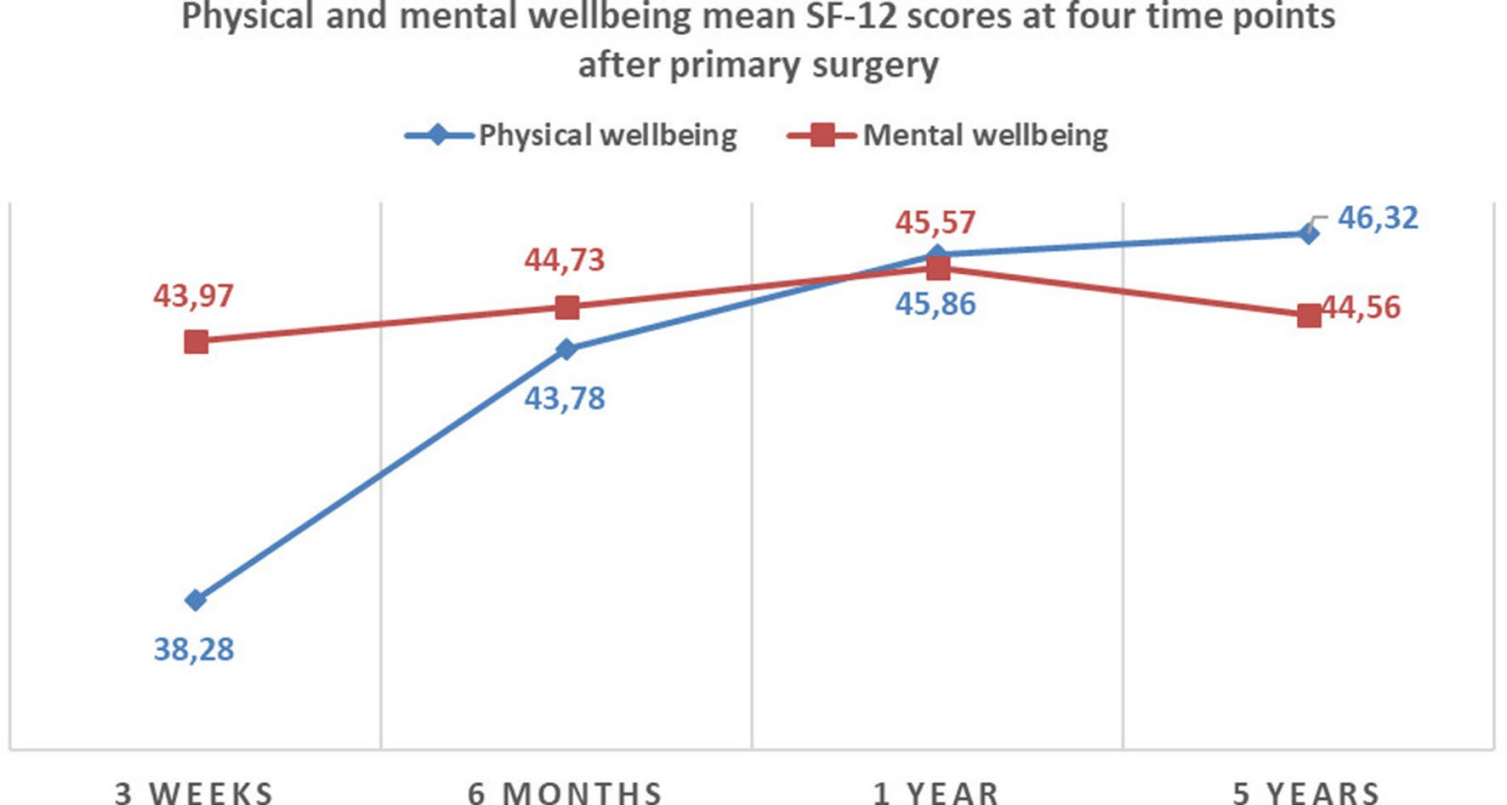
SF-12 mean scores for physical and mental wellbeing at 3 weeks, 6 months, 1 year and 5 years after primary surgery

The scale reliability coefficient Cronbach’s alpha was α = 0.90 for the first, second and fourth survey time points (3 weeks, 6 months and 5 years) and α = 0.86 for the third survey time point (1 year), indicating a satisfactory internal consistency of the SF-12 items [51]. SF-12 score for physical wellbeing at 3 weeks was 38.3 and rose by around 5.5 points at 6 months and 7.3 points at 1 year, indicating an improvement in this dimension of HRQL during the first year after primary surgery. However, there was barely any improvement in physical wellbeing between 1 year and 5 years after primary surgery. While SF-12 scores for mental wellbeing were higher than those for physical wellbeing at baseline, scores only increased slightly at 6 months (0,9 points) and at 1 year (1,6 points), and slightly deteriorated again at 5 years after primary surgery (Fig. 1).

### Factors associated with HRQL

After adjusting for other covariates that were significant at the univariate level (Table 2), the multivariate regression analysis showed that RTW was highly and significantly associated with better physical (Table 3) and mental wellbeing (Table 4) during the first year after primary surgical treatment. At 5 years, RTW was no longer a significantly associated factor. At this stage, income was significantly associated with physical (Table 3) and mental wellbeing. Moreover, living with the partner paired with income were positively associated factors with mental wellbeing, as significant effects were observed at 6 months and again at 5 years (Table 4). Among the examined therapies, chemotherapy was negatively associated with physical wellbeing at 6 months (Table 3), and antihormone therapy was negatively associated with mental wellbeing at 5 years (Table 4).

**Table 2:** Univariate linear regression analyses of sociodemographic and health related factors associated with physical and mental wellbeing at 3 weeks, 6 months, 1 year and 5 years after primary surgery

	Physical Wellbeing			Mental Wellbeing		
	Coef.	*p*	95% CI	Coef.	*p*	95% CI
Socio-Demographic Factors						
**Age**						
*3 weeks*	**-0.20**	**< 0.05**	-0.31, -0.08	0.03	0.65	-0.10, 0.16
*6 months*	-0.07	0.29	-0.20, 0.06	0.05	0.55	-0.11, 0.21
*1 year*	-0.13	0.06	-0.26, 0.01	0.05	0.50	-0.09, 0.19
*5 years*	0.02	0.79	-0.13, 0.16	0.08	0.24	-0.06, 0.22
**Education - Middle**						
*3 weeks*	1.20	0.39	-1.55, 3.95	0.65	0.69	-2.51, 3.82
*6 months*	1.18	0.45	-1.88, 4.24	-1.07	0.58	-4.91, 2.77
*1 year*	1.42	0.41	-1.93, 4.76	0.91	0.61	-2.62, 4.43
*5 years*	1.60	0.38	-2.00, 5.17	0.10	0.95	-3.39, 3.59
**Education - High**						
*3 weeks*	1.98	0.39	-1.55, 3.95	0.63	0.69	-2.52–3.78
*6 months*	**3.21**	**< 0.05**	0.14, 6.28	0.92	0.64	-2.93, 4.77
*1 year*	2.71	0.11	-0.59, 6.02	1.23	0.61	-2.62, 4.43
*5 years*	3.06	0.09	-0.45, 6.58	0.89	0.61	-2.55, 4.32
**Occupation - Middle autonomy** *(ref: Low autonomy)*						
*3 weeks*	0.17	0.87	-1.85, 2.19	0.46	0.70	-1.84, 2.75
*6 months*	1.27	0.27	-0.99, 3.53	-1.83	0.20	-4.62, 0.96
*1 year*	-0.03	0.98	-2.39, 2.33	-1.60	0.20	-4.07, 0.86
*5 years*	1.00	0.45	-1.55, 3.51	-0.81	0.52	-3.28, 1.66
**Occupation - High autonomy** *(ref: Low autonomy)*						
*3 weeks*	0.86	0.47	-1.47, 3.19	1.82	0.18	-0.82, 4.46
*6 months*	**4.67**	**< 0.001**	2.07, 7.26	1.43	0.38	-1.77, 0.96
*1 year*	2.01	0.14	-0.69, 4.72	-0.33	0.82	-3.15, 2.50
*5 years*	2.06	0.15	-0.78, 4.90	0.46	0.74	-2.30, 3.23
**Income**						
*3 weeks*	0.0003	0.68	-0.001, 0.002	**0.003**	**< 0.001**	0.002, 0.005
*6 months*	**0.003**	**< 0.001**	0.002, 0.004	**0.003**	**< 0.001**	0.001, 0.005
*1 year*	**0.002**	**< 0.05**	0.0008, 0.004	0.001	0.20	-0.0005, 0.002
*5 years*	**0.002**	**< 0.05**	0.0009, 0.004	**0.002**	**< 0.05**	0.0006, 0.003
**RTW**						
*3 weeks*	**6.69**	**< 0.001**	3.89, 9.50	**3.81**	**< 0.05**	0.54, 7.07
*6 months*	**7.66**	**< 0.001**	5.74, 9.58	**7.15**	**< 0.001**	4.73, 9.57
*1 year*	**10.65**	**< 0.001**	8.20, 13.11	**8.56**	**< 0.001**	5.89, 11.24
*5 years*	**3.63**	**< 0.05**	1.10, 6.15	1.41	0.27	-1.08, 3.90
**Living with partner**						
*3 weeks*	0.31	0.76	-1.65, 2.27	1.59	0.16	-0.63, 3.80
*6 months*	0.08	0.94	-2.08, 2.25	**4.14**	**< 0.05**	1.52, 6.77
*1 year*	0.51	0.65	-1.71, 2.73	-0.07	0.95	-2.40, 2.26
*5 years*	0.04	0.98	-2.49, 2.42	**3.79**	**< 0.05**	1.45, 6.14
**Migratory Background**						
*3 weeks*	-0.60	0.59	-2.81, 1.61	**-3.91**	**< 0.05**	-6.38, -1.44
*6 months*	-0.75	0.55	-3.21, 1.70	**-3.5**	**< 0.05**	-6.51, -0.48
*1 year*	**-3.06**	**< 0.05**	-5.56, -0.55	0.13	0.92	-2.51, 2.77
*5 years*	-1.84	0.19	-4.59, 0.90	0.45	0.74	-2.23, 3.12
**Health-related factors**						
**UICC − 2** *(ref: 1)*						
*3 weeks*	-1.00	0.28	-2.81, 0.80	0.28	0.79	-1.78, 2.33
*6 weeks*	-1.02	0.33	-3.06, 1.03	-1.37	0.28	-3.88, 1.14
*1 year*	-0.74	0.48	-2.82, 1.34	-0.77	0.49	-2.96, 1.42
*5 years*	**-3.52**	**< 0.05**	-5.72, -1.31	-1.48	0.18	-3.66, 0.70
**UICC − 3** *(ref: 1)*						
*3 weeks*	**-3.57**	**< 0.05**	-6.76, -0.37	1.49	0.42	-2.14, 5.13
*6 weeks*	-1.16	0.53	-3.06, 1.03	-0.84	0.71	-5.30, 3.62
*1 year*	-3.45	0.06	-7.08, 0.18	0.17	0.93	-3.64, 1.42
*5 years*	**-5.24**	**< 0.05**	-9.23, -1.26	-1.2	0.55	-5.13, 2.74
**Neoadjuvant Chemotherapy**						
*3 weeks*	**-2.86**	**< 0.05**	-3.84, -0.33	1.98	0.05	-0.02, 3.97
*6 weeks*	0.28	0.78	-1.72, 2.29	1.82	0.15	-0.63, 4.27
*1 year*	0.66	0.52	-1.37, 2.69	1.17	0.28	-0.95, 3.30
*5 years*	-0.15	0.90	-2.36, 2.06	0.2	0.86	-1.95, 2.34
**Chemotherapy**						
*3 weeks*	*N.A.*					
*6 weeks*	**-7.18**	**< 0.001**	-9.35, -5.01	-2.4	0.09	-5.18, 0.39
*1 year*	**-4.78**	**< 0.05**	-7.70, -1.86	-1.87	0.24	-4.97, 1.22
*5 years*	**-9.48**	**< 0.05**	-16.45, -2.49	-6.03	0.08	-12.86, 0.79
**Radiation therapy**						
*3 weeks*	*N.A.*					
*6 weeks*	1.33	0.22	-0.81, 3.47	1.79	0.18	-0.83, 4.41
*1 year*	**-3.34**	**< 0.05**	-5.69, -0.99	-2.24	0.08	-4.72, 0.25
*5 years*	**-10.5**	**< 0.05**	-19.32, -1.69	-2.18	0.62	-10.81, 6.45
**Antihormone therapy**						
*3 weeks*	*N.A.*					
*6 weeks*	**2.23**	**< 0.05**	0.29, 4.16	0.73	0.55	-1.66, 3.11
*1 year*	-0.04	0.97	-2.08, 2.00	-0.77	0.48	-2.90, 1.37
*5 years*	-1.84	0.09	-3.94, 0.27	**-2.31**	**< 0.05**	-4.35, -0.27
**Mastectomy** *(ref: BCT)*						
*3 weeks*	**-3.16**	**< 0.05**	-5.19, -1.13	1.77	0.13	-0.53, 4.07
*6 weeks*	-0.5	0.67	-2.79, 1.80	-0.16	0.91	-2.97–2.65
*1 year*	-0.94	0.44	-3.33, 1.44	-0.37	0.77	-2.88, 2.13
*5 years*	-2.49	0.06	-5.08, 0.10	-1.91	0.14	-4.43, 0.61
**Rehabilitation therapy**						
*3 weeks*	*N.A.*					
*6 weeks*	1.21	0.22	-0.74, 3.16	-0.72	0.56	-3.11, 1.67
*1 year*	**-5.22**	**< 0.001**	-7.42, -3.02	**-3.99**	**< 0.05**	-6.33, -1.65
*5 years*	**-8.73**	**< 0.05**	-16.20, -1.25	-6.4	0.08	-13.69, 0.87
**Membership in self-help group**						
*3 weeks*	*N.A.*					
*6 weeks*	-2.83	2.04	-6.85, 1.19	-2.49	0.32	-7.42, 2.44
*1 year*	**-6.65**	**< 0.05**	-11.40, -1.90	**-6.80**	**< 0.05**	-11.78, -1.82
*5 years*	-1.24	0.39	-4.06, 1.58	0.23	0.87	-2.51, 2.96

**Table 3:** Multivariate linear regression analyses on factors associated with physical wellbeing at 3 weeks, 6 months, 1 year and 5 years after primary surgery

	Coef.	*P* value	95% CI
3 weeks			
**Age**	**-0.22**	**< 0.001**	-0.34, -0.10
**RTW**	**6.21**	**< 0.001**	3.36, 9.05
**UICC** *(ref: 1)*			
*2*	-0.14	0.88	-1.96, 1.68
*3*	-2.06	0.24	-5.51, 1.38
**Mastectomy** *(ref: BCT)*	**-2.49**	**< 0.05**	-4.67, -0.30
**Neoadjuvant chemotherapy**	-1.67	0.07	-3.50, 0.16
**6 months**			
**RTW**	**5.40**	**< 0.001**	3.01, 1.80
**Income**	0.001	0.09	-0.0002, 0.003
**Occupational position**			
*middle autonomy*	-0.94	0.51	-3.74, 1.86
*high autonomy*	1.13	0.56	-2.64, 4.89
**Education**			
*middle*	0.86	0.65	-2.89, 4.62
*high*	1.22	0.55	-2.80, 5.24
**Chemotherapy**	**-4.25**	**< 0.05**	-7.04, -1.46
**Antihormone therapy**	-0.48	0.67	-2.75, 1.78
**1 year**			
**RTW**	**8.40**	**< 0.001**	5.31, 11.49
**Income**	0.001	0.13	-0.0003, 0.003
**Radiation therapy**	0.39	0.82	-3.00, 3.78
**Chemotherapy**	-0.79	0.70	-5.35, 0.14
**Migratory background**	-2.60	0.06	-5.35, 0.14
**Rehabilitation therapy**	-0.89	0.52	-3.65, 1.87
**Membership in self-help group**	-3.92	0.14	-9.12, 1.27
**5 years**			
**RTW**	2.58	0.06	-0.14, 5.31
**Income**	**0.002**	**< 0.05**	0.0002, 0.003
**Radiation therapy**	-1.20	0.83	-12.35, 9.94
**Chemotherapy**	-4.06	0.32	-12.07, 3.96
**UICC** *(ref: 1)*			
*2*	**-3.73**	**< 0.05**	-6.26, -1.20
*3*	**-5.42**	**< 0.05**	-9.92, -0.93
**Rehabilitation therapy**	-7.94	0.07	-16.53, 0.65

3 weeks: *N* = 368 ; 6 months: *N* = 286; 1 year: *N* = 261; 5 years: *N* = 255

**Table 4:** Multivariate linear regression analyses on factors associated with mental wellbeing at 3 weeks, 6 months, 1 year and 5 years after primary surgery

	Coef.	*P* value	95% CI
3 weeks			
**RTW**	1.99	0.26	-1.50, 5.47
**Income**	**0.003**	**< 0.001**	0.001, 0.004
**Migratory background**	-2.73	0.05	-5.47, 0.009
**6 months**			
**RTW**	**6.03**	**< 0.001**	3.25, 8.81
**Income**	**0.002**	**< 0.05**	0.002, 0.004
**Living with partner**	**3.68**	**< 0.05**	0.72, 6.63
**Migratory background**	-2.06	0.24	-5.47, 1.35
**1 year**			
**RTW**	**7.71**	**< 0.001**	4.85, 10.58
**Rehabilitation therapy**	-1.48	0.23	-3.92, 0.95
**Membership in self-help group**	-2.86	0.26	-7.89–2.16
**5 years**			
**Income**	**0.002**	**< 0.05**	0.0005, 0.003
**Living with partner**	**2.85**	**< 0.05**	0.39, 5.32
**Antihormone therapy**	**-2.23**	**< 0.05**	-4.37, -0.1

3 weeks: *N* = 301; 6 months: *N* = 276; 1 year: *N* = 333; 5 years: *N* = 295

### Vulnerabilities in HRQL

#### 3 Weeks

**Fig. 2:**
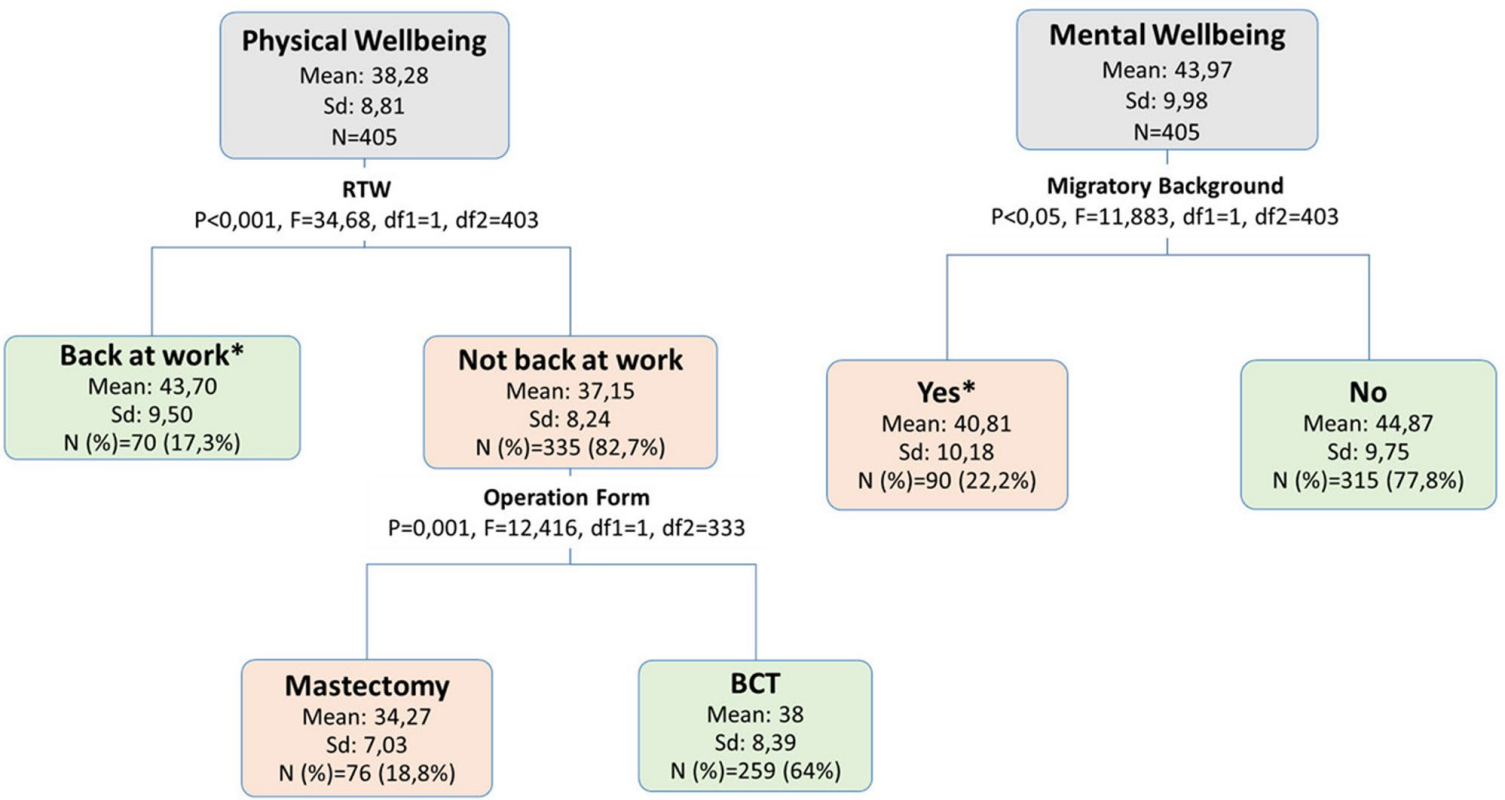
Decision tree analysis of factors that define vulnerable groups with respect to physical and mental wellbeing at 3 weeks after primary surgery. *Missing values were grouped into this node

At this time point, RTW appeared to be the factor mostly correlated with physical wellbeing. Those who were back at work at this time point had a mean SF-12 score for physical wellbeing of around 44, compared with a mean score of 37 in women who were not back at work. The fact of having had a mastectomy compared to BCT interacted significantly to be a second level associated factor with a more deteriorated physical wellbeing: among those who did not RTW at this time point, mastectomy was associated with around four points lower in the SF12 score for physical wellbeing compared to BCT. With respect to mental wellbeing, the first splitting factor was having a migratory background with a negative contribution and around four score points lower in the mental wellbeing SF12 score compared to women with no migratory background. No other factors appeared to be correlated at a next level (Fig. 2).

#### 6 Months

**Fig. 3:**
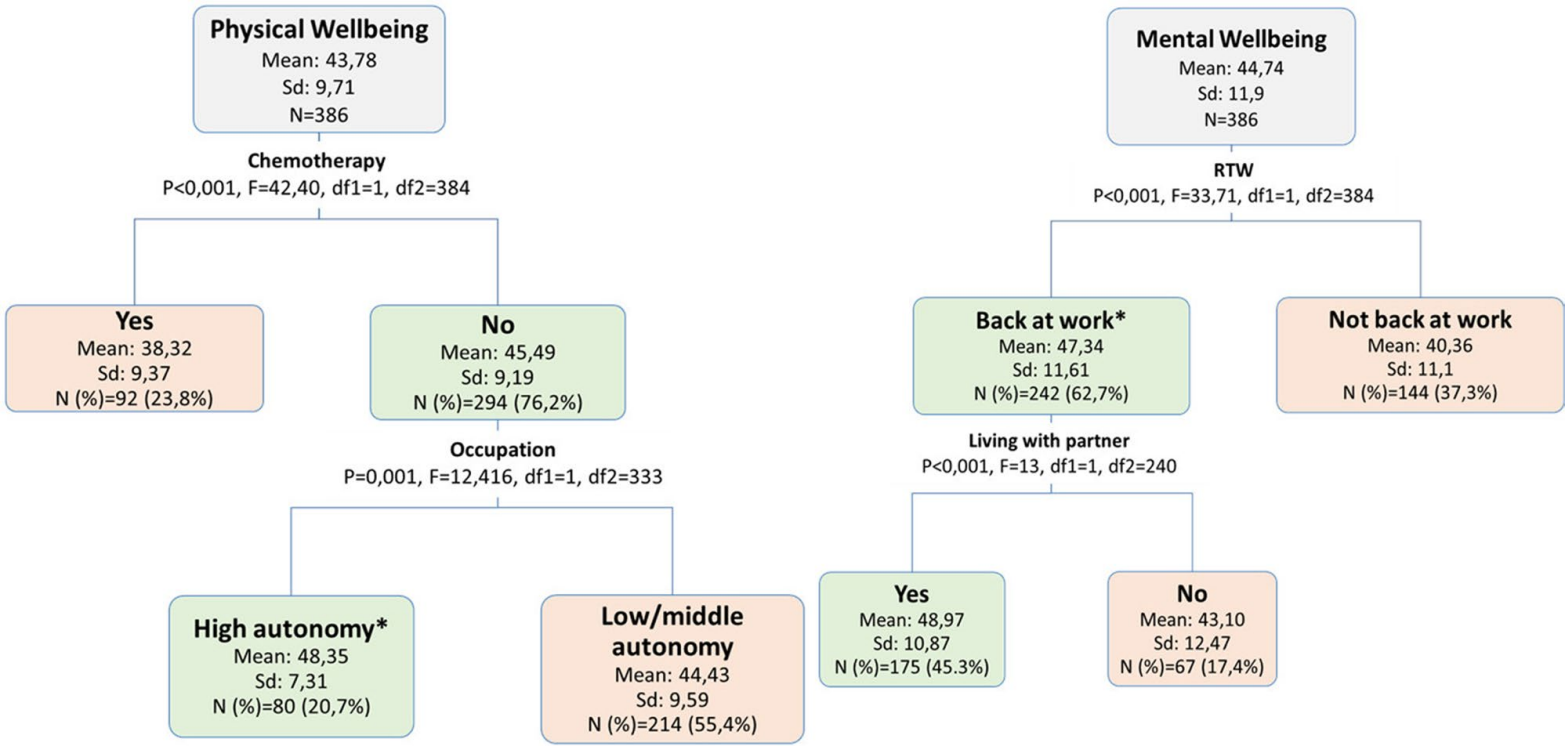
Decision tree analysis of factors that define vulnerable groups with respect to physical and mental wellbeing at 6 months after primary surgery. *Missing values were grouped into this node

At this time point, chemotherapy was the mostly associated factor with physical wellbeing with around seven SF12 mean score points higher for those who have not had a chemotherapy during the past six months. At the second level, occupation interacted significantly in the cluster of women who did not have a chemotherapy, with significantly higher SF12 score for physical wellbeing for individuals with high autonomy occupations. With respect to mental wellbeing, RTW was the mostly associated factor at this time point, with around seven score points higher for the group of women who were back at work. Among those who were back at work, living with the partner was associated with a better mental wellbeing with around eight score points higher compared to those not living with a partner (Fig. 3).

#### 1 Year

**Fig. 4:**
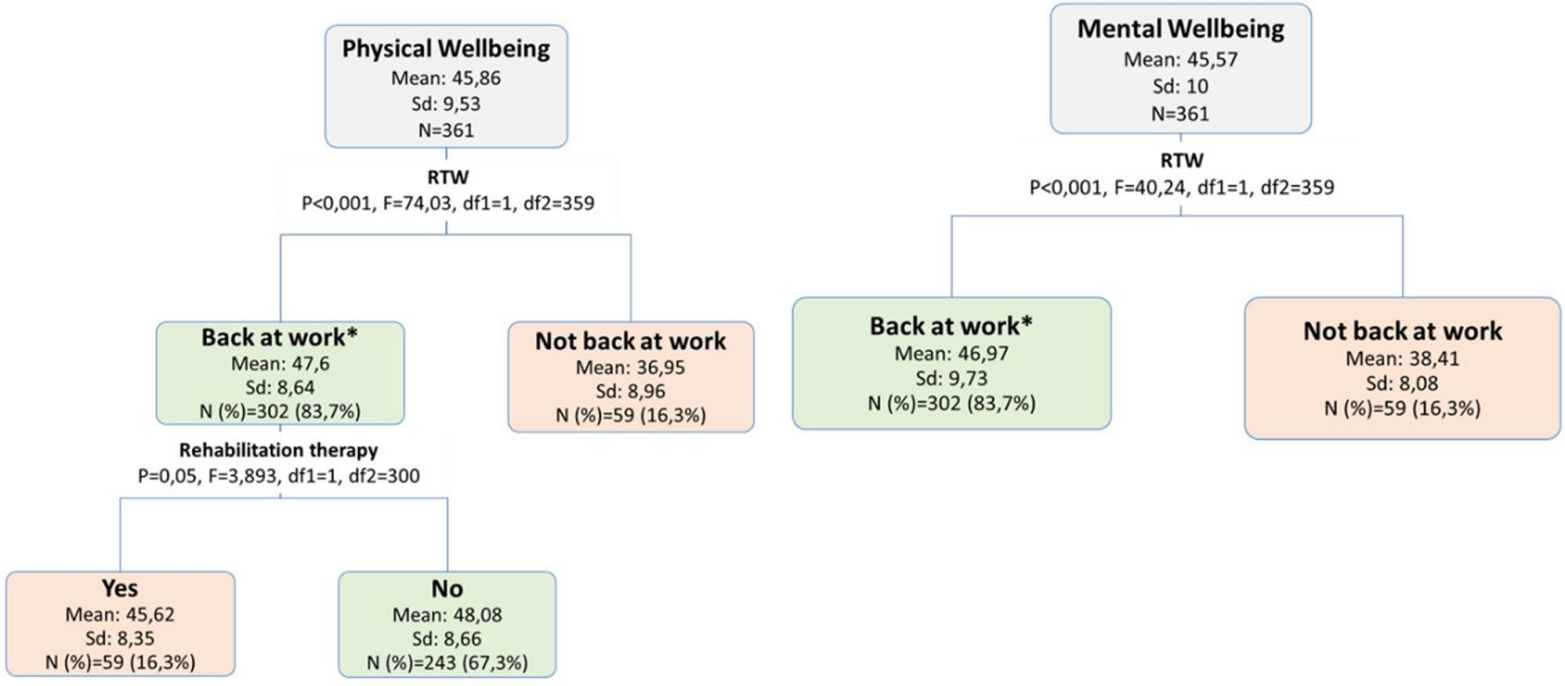
Decision tree analysis of factors that define vulnerable groups with respect to physical and mental wellbeing at 1 year after primary surgery. *Missing values were grouped into this node

Around one year after primary surgery, occupation and chemotherapy were no more associated with HRQL. At this time period, being back at work was the leading associated factor with physical and mental wellbeing (around nine points higher in the mean scores). For women who were back at work, having had rehabilitation therapy during the last six months was negatively associated with physical wellbeing, but was only borderline significant (Fig. 4).

#### 5 Years

**Fig. 5:**
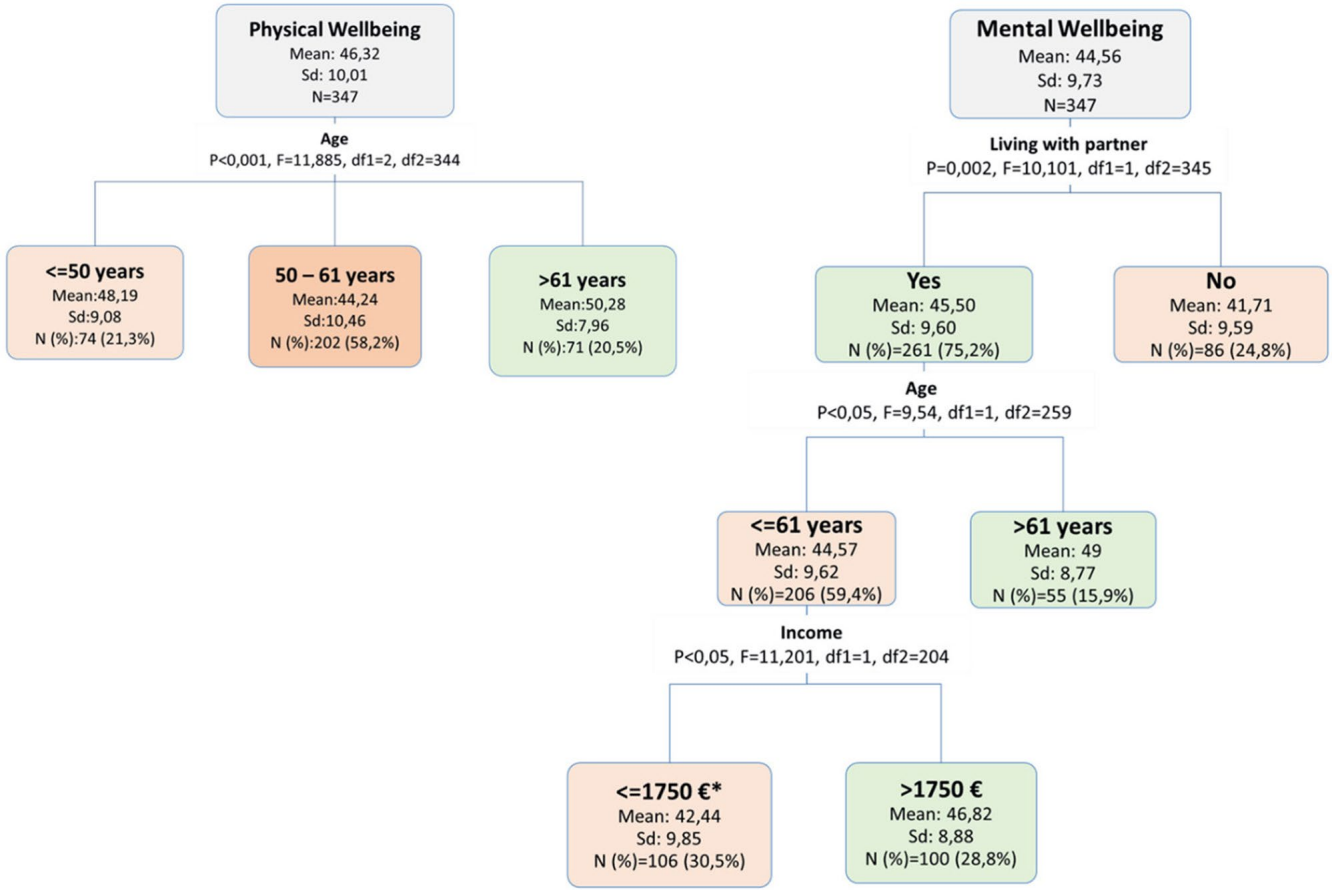
Decision tree analysis of factors that define vulnerable groups with respect to physical and mental wellbeing at 5 years after primary surgery. *Missing values were grouped into this node

At this stage, RTW was no longer associated with physical and mental wellbeing. At this stage, age was the most important splitting for both measures of HRQL. The cut-off age was splitted by the decision tree analyses at 50 years and 61 years. Women aged 50–61 years had the lowest mean scores (Mean = 44), followed by the age group < = 50 years (Mean = 48), and the age group > 61 years (Mean = 50). With respect to mental wellbeing, living with the partner was the most important splitting factor. Among those living with a partner at this time period, women aged > 61 years had also better mental wellbeing scores compared to women < = 61 years. In this cluster, additional factors were significantly associated: income had a synergetic effect on better mental wellbeing, where women with an income of > 1750€ had higher mental wellbeing scores (Fig. 5).

## Discussion

This study examined HRQL of women with breast cancer during the first five years after diagnosis, with a close observation of several time points during the first years after primary surgery. SF-12 scores were calculated for physical and mental wellbeing at 3 weeks, 6 months, 1 year and on average 5 years after primary surgery. Compared with a norm sample from the German national health survey for which the same SF-12 version was used [39], SF-12 scores for physical and mental wellbeing in our sample are considerably lower at 3 weeks and 6 months after primary surgery, and remain to be lower than the norm sample at 1 year and at 5 years, especially for mental wellbeing. When filtering the German norm sample to include women with age groups that match those of our sample, it will consist of 2988 disease-free women with the following mean SF-12 scores: 47.87 for physical wellbeing and 49.47 for mental wellbeing. While these scores are relatively outdated, and recent age and gender stratified mean scores are not available for comparison, the results might indicate that physical and mental wellbeing are deteriorated after breast cancer diagnosis, which remained to be the case at 5 years especially for mental wellbeing. This conclusion is consistent with the results of numerous studies that found an adverse effect of breast cancer on HRQL [13, 18, 47].

### Factors associated with HRQL

The study investigated factors associated with HRQL in women with breast cancer at four time points during the first 5 years after primary surgery. This was done using two methods, the first one entailing a classical multivariate regression analysis including variables that were shown to be significant at the univariate level. The second method entailed regression tree analysis that simultaneously included all examined variables in the models, which was done to define specific vulnerable groups by considering intersectional vulnerabilities with respect to the considered variables, which was not to be revealed using the classical regression method. The two analyses revealed mostly similar results with respect to the significantly associated factors, but more specific vulnerable subgroups could be defined using the tree method. The study showed that HRQL in women with breast cancer is a multifaceted issue associated with disease, type of treatment and sociodemographic factors.

Among other examined factors, RTW was shown to be strongly and positively associated with HRQL during the first year after primary surgery, while ranking as the first splitting factor in decision trees for most of the outcomes. Previous research has examined the association between RTW after breast cancer and HRQL. A systematic review of 28 studies concluded that being employed and missing fewer days at work are associated with better physical and mental wellbeing in breast cancer survivors [24]. Employment is associated with better body image and physical functioning [19], while research suggests that being on sick leave was significantly associated with a lower HRQL among breast cancer survivors [25]. Moreover, not only working status, but also the number of hours worked per week has been shown to be positively associated with quality of life among breast cancer survivors [48]. In addition to its predicting effect on quality of life, employment status among breast cancer survivors has been significantly associated with higher 4-years survival rate [49]. Moreover, evidence suggests that deteriorated mental and social wellbeing are associated with disease recurrence and a poorer prognosis among women with breast cancer [50, 51]. The observed positive association between having returned to work and HRQL in breast cancer patients may thus be explained through better social interaction and distraction from their disease. However, the association between RTW and HRQL could be also demonstrated in a different direction. Individuals with a higher HRQL might be more likely to RTW due to better physical readiness. In order to rule out or confirm this possibility, the direction of association between RTW and HRQL should be examined. Confirming the concluded direction of association (RTW ◊ better HRQL) would highlight the importance of promoting RTW after breast cancer even in the early treatment phase when they are trying to cope with their new situation.

Nevertheless, at 5 years after surgery, RTW was no longer a significantly associated factor, nor was a high-ranking splitting factor that defines vulnerable groups with respect to HRQL. At this stage, among the vulnerable groups were women aged < 61 years old, with the lowest physical wellbeing mean score for the age group 50–61 years. Age was also a splitting factor for mental wellbeing with better mean scores for women aged > 61 years. While the mentioned age groups with lower physical and mental wellbeing mean scores might be generally vulnerable groups regardless of breast cancer, among breast cancer patients, the process of ageing may have an impact on the ability to accommodate and to adjust with disease and treatment side effects. In fact, several studies showed age to be a significant predictor for quality of life in breast cancer patients [52, 53]. Nevertheless, the association between age and HRQL did not appear to be linear in this study, as women aged 50–61 appeared to be more vulnerable than younger and older women. In fact, this is in line with the evidence that suggests that subjective health improves after pension, as individuals tend to experience a relief from many responsibilities associated with being employed [54]. Moreover, the vulnerable age group is associated with a peak of menopausal symptoms that could have a significant effect on HRQL. Thus, a special focus should be drawn on the specified vulnerable age group when designing strategies that aim at improving HRQL in breast cancer survivors.

Among the vulnerable groups with respect to mental wellbeing were women not living with a partner. At 5 years post surgery for example, women who were not living with a partner had the lowest mean scores for mental wellbeing among the child nodes (M = 41). This finding was also evident in other studies where it was shown that women recovering from breast cancer who are married or living with a partner had better HRQL, which was mediated through higher levels of social support [55]. In another study that investigated perceived social support in women with breast cancer, the spouse or partner was observed by the vast majority of the sample as being the most important supporter while having a central role during the recovery phase [56]. Thus, rehabilitation programs could consider incorporating partners in programs that aim at improving support systems for breast cancer patients during their therapeutic and recovery journey. Moreover, a special focus should be drawn to women not living with a partner, as they appear to be a vulnerable group with respect to mental wellbeing.

Among the health related factors investigated, mastectomy and chemotherapy at 3 weeks and 6 months after surgery respectively were among the mostly associated factors HRQL as illustrated by lower scores for physical wellbeing. This is expected as these treatments cause uncomfortable side effects such as pain, nausea, fatigue, hair loss and others, and their negative effects on quality of life have been well established [20, 57, 58]. However, this study showed that their effect was no more apparent at 1 year and later. Supporting results have been illustrated in another study investigating the effect of chemotherapy on quality of life in breast cancer. It was shown that chemotherapy had a significantly negative effect on quality of life at 6 months after diagnosis, but this effect resolves by 18 months [59]. Similarly, there is evidence that the negative effect of mastectomy on quality of life diminishes by time to be equal to the effect of having undergone a breast conserving surgery [60]. Thus, these results might be important to provide evidence for early-stage breast cancer patients who might be confronted with decision making on treatment choices.

Among the socioeconomic factors, income appeared to be critical with respect to HRQL, especially for mental wellbeing during the early phase after primary surgery and again at 5 years while showing its effect also on physical wellbeing at this stage. In addition, the specific subgroup of women aged ≤ 61 years with lower income was shown to be vulnerable with respect to mental wellbeing at 5 years. While the effect of income on HRQL in general has been established [61], our results also point towards the significant effect of income in determining HRQL in breast cancer, especially with respect to mental wellbeing. Nevertheless, there is evidence on the mediating effect of income on the association between job loss and wellbeing in breast cancer survivors [62]. Against the background of the results mentioned above on the strong positive association between RTW and HRQL, it should be examined in further analyses whether this association could be mediated by income.

### Practical implications

In Germany, medical and occupational rehabilitation are considered in the national guideline for cancer treatment as well as in the National Cancer Plan and have become a firmly established component in the care of cancer patients. The oncological rehabilitation includes thirteen evidence-based therapy modules with mostly a physical but also some psychosocial emphasis. The rehabilitation of breast cancer patients is almost exclusively inpatient with a duration of three to four weeks [63]. However, this duration might not be sufficient to address and deal with HRQL issues following breast cancer. In addition, there is evidence that many patients fail to receive sufficient psycho-oncological care [64]. Moreover, a thorough review of the literature indicates that a greater focus is drawn to the physical wellbeing outcomes of rehabilitation and support measures [65–67]. Therefore, results of this study highlight the importance of emphasizing the mental wellbeing dimension of HRQL in measures offered for breast cancer survivors while considering adequate accessibility, duration and aftercare in order to ensure much needed support.

While this study showed that RTW is strongly associated with a better HRQL during the first year after primary surgical treatment, the question remains open for further research to investigate whether the association between RTW and HRQL is causal (RTW leads to better HRQL) or selective (those with better HRQL are more likely to RTW). Still, research suggests that while the causal association might be more significant, both directions are interrelated and underpin each other [68, 69]. Therefore, ensuring the adequate planning and application of occupational rehabilitation measures that ease RTW (29–31) would be necessary. In addition, breast cancer survivors should be encouraged by health care providers to take advantage of the available medical and occupational rehabilitation services in order to facilitate their return to normal life and work as early as possible.

### Limitations

Results of this study should be considered in light of several limitations. Even though the study involved a complete recruitment of certified breast cancer centers in Hannover, there is an underestimation of rural areas since only two certified breast cancer centers were included in the study from rural regions. In addition, the results of this study are only generalizable to employed women with breast cancer. Even though response rate was relatively high (84%), the possible existence of dropout bias cannot be ruled out assuredly since very ill patients would be less likely to participate [70]. However, a sensitivity analysis was performed examining SF-12 sum scores for physical and mental wellbeing at the four time points of the study for only the women who remained till the end of the study, and very minimal differences were observed compared to the whole sample (supplement file 2). Since the outcome and most of the independent variables in this study are self-reported, there is a possible existence of reporting bias. In addition, the study only considered baseline occupational autonomy with the assumption that it would not change over the study period, especially in late work life [71]. However, this cannot be ruled out completely.

## Conclusion

The results of this study revealed that HRQL in breast cancer is a multidimensional phenomenon that is associated with the disease, type of therapy as well as social factors. Among the associated factors with HRQL, RTW is one that is modifiable and was shown to be the strongest associated factor. Nevertheless, examining the direction of associations between RTW and HRQL is central for confirming its predicting effect. In this case, promoting RTW after breast cancer, especially through providing adequate occupational support, would have a positive influence on HRQL. A special focus should be drawn to specific vulnerable groups, such as women with a migratory background, those not living with a partner, or those in the age group of 50–61 years when planning and implementing strategies aimed at improving HRQL in breast cancer.

## Electronic supplementary material

Below is the link to the electronic supplementary material.

Supplementary Material 1

Supplementary Material 2

## Data Availability

The datasets used and analyzed in this study are available from the corresponding author on reasonable request.

## Abbreviations

HRQL: Health-related quality of life
RTW: Return to work
SF-12: Short form health survey-12
OECD: Organization for economic cooperation and development
UICC: Union for international cancer control
BCT: Breast conserving treatment
