# Supplementary material for: Factors associated with health-related quality of life in women with paid work at breast cancer diagnosis: a German repeated cross-sectional study over the first five years after primary surgery
Source: BMC Cancer. 2025 Jan 17;25:98. doi: 10.1186/s12885-025-13491-8 (PMC11745005; doi:10.1186/s12885-025-13491-8)
Supplement: Supplementary file 1 — Supplementary Material 1 [file 12885_2025_13491_MOESM1_ESM.docx]

**Factors associated with health related quality of life in women with paid work at breast cancer diagnosis: A German repeated cross-sectional study over the first five years after primary surgery**

# First questionnaire: 3 weeks after primary surgery

### Are you currently employed? What applies to you?

*If you are currently on maternity leave, this is considered as employment*

**I am…**

| working fulltime | O |  |
| --- | --- | --- |
| working parttime | O |  |
| in occupational training, apprentice, or retraining | O |  |
| marginally or irregularly employed | O |  |
| in partial retirement with zero working time | O |  |
| Not working | O |  |
| other: ____________________________________________________ | O |  |

**Thank you for your willingness to participate and your cooperation so far. However, since this study only includes employed persons, we ask you to return the empty questionnaire to us. Thank you so much!**

### What is your current occupation?

### ***If you have more than one occupation, please answer the following questions only for your current main occupational activity.***

| **Worker (also in agriculture)** |  |  | **Civil servant (including judge and soldier)** | |
| --- | --- | --- | --- | --- |
| Unskilled worker | O |  | Lower level | O |
| Semi-skilled worker | O |  | Middle level | O |
| Trained worker or skilled worker | O |  | Upper level | O |
| Forewoman, Column leader | O |  | Executive level | O |
| Master craftsperson | O |  |  |  |
|  |  |  | **Self employed** |  |
| **Employee** |  |  | Independent farmer | O |
| Employee with simple tasks and no qualifications | O |  | Free profession, self-employed academic | O |
| Employee with simple tasks  with a qualification | O |  | Other | O |
|  |  |  | Family worker | O |
| Employed master | O |  | **Apprentice** |  |
| Employee with qualified work (e.g. clerk, technical draftswoman) | O |  | In vocational training | O |
|  |  |  | In internship | O |
| Employees with a highly qualified job or managerial function (e.g. research assistant, engineer, department head) | O |  | Student | O |
|  |  |  | **Other: ________________** | O |

### Are you currently on sick leave?

No O Yes O

### Were you on sick leave during the last 12 months?

| No | O | I am self employed | O | Yes | O | Total: |  |  | Weeks |  | Days |
| --- | --- | --- | --- | --- | --- | --- | --- | --- | --- | --- | --- |

### Which family status do you have?

| Married and living with my husband | O |
| --- | --- |
| In a registered partnership, living together (same sex) | O |
| Married and living separately from my husband | O |
| Single | O |
| Divorced | O |
| Widowed | O |
| In a registered partnership, not living together (same sex) | O |
| Ended registered partnership (same sex) | O |
| Registered partner died (same sex) | O |

### Are you in a firm relationship?

No O🡪 go to question 37! Yes O Yes, same sex O

### Does your partner live at your house?

No O Yes O

### How many individuals live at your house?

| 1 | 2 | 3 | 4 | 5 | 6 | 7 | 8 | More than 8 |
| --- | --- | --- | --- | --- | --- | --- | --- | --- |
| O | O | O | O | O | O | O | O | O |

### How many individuals living at your house are under 18 years old?

| 0 | 1 | 2 | 3 | 4 | 5 | 6 | 7 | More than 7 |
| --- | --- | --- | --- | --- | --- | --- | --- | --- |
| O | O | O | O | O | O | O | O | O |

### Since when have you been mainly living in Germany?

| Since birth | O | Since (number of years) |  |
| --- | --- | --- | --- |

### Were both of your parents born in Germany?

### ***Germany with its actual borders is meant here.***

No O Yes O 🡪 Go to question 43!

| In which country was your mother born? | __________________________ |
| --- | --- |
| In which country was your father born? | __________________________ |

### What is your highest achieved school certificate?

| Student, attending a full-time general education school | O |
| --- | --- |
| Left school without achieving secondary general school | O |
| Secondary general school (Hauptschule) | O |
| Intermediate secondary school (Realschule) | O |
| Polytechnic high school (GDR), 8^th^ or 9^th^ grade | O |
| Polytechnic high school (GDR), 10^th^ grade | O |
| Technical high school (Fachoberschule” (Qualification for studies at a technical college) | O |
| Upper secondary school (Abitur) | O |
| Upper secondary school (Abitur) achieved at evening school for adults | O |
| Another school certificate, and that is:_______________________________ | O |

### What is your highest achieved vocational training qualification?

### ***Please provide only one answer!***

| Apprenticeship, skilled worker certificate | O |
| --- | --- |
| Vocational school for crafts / trades / agriculture / health sector | O |
| Vocational school such as a school for master craftspeople or technical college | O |
| Civil servant training | O |
| Technical college / school of engineering | O |
| University degree | O |
| Doctoral degree | O |
| Other | O |

### What is the total monthly net income of your household?

### *These include the wages or incomes of all people living and working in the household, as well as rental income, child benefit, parental benefit, unemployment benefit, Hartz IV, social benefit, accommodation costs, housing benefit, pension, etc.*

| under 750 € | 750 to under 1250 € | 1250 to under 1750 € | 1750 to under 2250 € | 2250 to under 3000 € | 3000 to under 4000 € | |
| --- | --- | --- | --- | --- | --- | --- |
| O | O | O | O | O | O | |
| 4000 to under 5000 € | 5000 € and more | I don’t want to answer | I don’t know |  |  | |
| O | O | O | O |  |  |  |

# Second Questionnaire: 6 months after primary surgery

### Are you currently employed? What applies to you?

*If you are currently on maternity leave, this is considered as employment*

**I am…**

| working fulltime | O |
| --- | --- |
| working parttime | O |
| marginally or irregularly employed | O |
| self-employed/independent | O |
| in a permanent position | O |
| in occupational training, apprentice, or retraining | O |
| in gradual occupational reintegration | O |
| working for another employer (compared to six months ago) | O |
| switched to self-employment | O |
| in a measure offered from the employment agency | O |
| unemployed | O |
| in partial retirement with zero working time | O |
| in pension | O |
| in an internship | O |
| on maternity leave | O |
| other: ____________________________________________________ | O |

### If you have completed a further school education or apprenticeship or your professional position has changed in the last 6 months, please make a note of this:

________________________________________________________________________________________________________________________________________________________________________________________________________________________________________________

### Were you during the past six months on sick leave (counted from now backwards)?

| No | O | I am self employed | O | Yes | O | In total: |  |  | Weeks |  | Days |
| --- | --- | --- | --- | --- | --- | --- | --- | --- | --- | --- | --- |

### Are you currently on sick leave? ***(Please answer all that applies!)***

| No | O |  |
| --- | --- | --- |
| Yes, due to a short-term illness | O |  |
| Yes, because of breast cancer I am not yet back at work | O | 🡪 Please go to question 13 |
| Yes, due to another long (chronic) illness, I have not been at work for months | O | 🡪 Please go to question 13 |
| For another reason, and that is: __________________________________________________________ | | |

### If you have returned to your job or intend to return, what are your reasons for doing so?

### *Non-returnees please tick “Does not apply”* O *Does not apply*

*Please tick an answer in each row!*

| **I return…** | Fully disagree | Agree a little | Moderately agree | Quite agree | | Fully agree | |
| --- | --- | --- | --- | --- | --- | --- | --- |
| because I want to live as I did before the disease | O | O | O | O | O | |  |
| because I am dependent on my earned income | O | O | O | O | O | |  |
| because I enjoy my work | O | O | O | O | O | |  |
| because I need the gainful employment for my pension | O | O | O | O | O | |  |
| because the social environment is important to me | O | O | O | O | O | |  |
| because I feel uncomfortable at home | O | O | O | O | O | |  |
| so that I would have a task | O | O | O | O | O | |  |
| because my daily routine is important to me | O | O | O | O | O | |  |
| Other, and that is: ______________________________________________________________ | | | | | | |  |

### If you are considering not returning to work or have already decided to quit, what are the reasons?

### *Returnees please tick “Does not apply”* O *Does not apply*

*Please tick an answer in each row!*

| **I will (probably) not return…** | Fully disagree | Agree a little | Moderately agree | Quite agree | Fully agree | |
| --- | --- | --- | --- | --- | --- | --- |
| because I lack the physical strength for it | O | O | O | O | O | |
| because I lack the nervous strength for it | O | O | O | O | O | |
| because there are more important things in life | O | O | O | O | O | |
| so that I have more time for my family/friends | O | O | O | O | O | |
| because I do not enjoy work | O | O | O | O | O | |
| because I have no financial need | O | O | O | O | O | |
| I can use my free time reasonably without gainful employment | O | O | O | O | O | |
| I would like to take a break in my professional career | O | O | O | O | O | |
| Other, and that is:_______________________________________________________________ | | | | | |  |

***The following questions relate to the period after discharge from the hospital with regard to further therapy and support services.***

### What treatments have you received within the last six months following your breast cancer surgery? ***(Multiple selections possible!)***

| Further operations on the same  or other breast | O | Other drug treatments | O |
| --- | --- | --- | --- |
| Breast reconstruction | O | alternative practitioners/alternative medicine | O |
| Radiation therapy | O | Rehab/follow-up treatment | O |
| Chemotherapy | O | Self-help group | O |
| Psychotherapy | O | None | O |
| Antihormone therapy | O | Other___________________________ |  |

### Which family status do you have?

| Married and living with my husband | O |
| --- | --- |
| In a registered partnership, living together (same sex) | O |
| Married and living separately from my husband | O |
| Single | O |
| Divorced | O |
| Widowed | O |
| In a registered partnership, not living together (same sex) | O |
| Ended registered partnership (same sex) | O |
| Registered partner died (same sex) | O |

### Are you in a firm relationship?

No O🡪 go to question 44! Yes O Yes, same sex O

### Does your partner live at your house?

No O Yes O

### How many individuals live at your house?

| 1 | 2 | 3 | 4 | 5 | 6 | 7 | 8 | More than 8 |
| --- | --- | --- | --- | --- | --- | --- | --- | --- |
| O | O | O | O | O | O | O | O | O |

### How many individuals living at your house are under 18 years old?

| 0 | 1 | 2 | 3 | 4 | 5 | 6 | 7 | More than 7 |
| --- | --- | --- | --- | --- | --- | --- | --- | --- |
| O | O | O | O | O | O | O | O | O |

### What is the total monthly net income of your household?

### *These include the wages or incomes of all people living and working in the household, as well as rental income, child benefit, parental benefit, unemployment benefit, Hartz IV, social benefit, accommodation costs, housing benefit, pension, etc.*

| under 750 € | 750 to under 1250 € | 1250 to under 1750 € | 1750 to under 2250 € | 2250 to under 3000 € | 3000 to under 4000 € | |
| --- | --- | --- | --- | --- | --- | --- |
| O | O | O | O | O | O | |
| 4000 to under 5000 € | 5000 € and more | I don’t want to answer | I don’t know |  |  | |
| O | O | O | O |  |  |  |

# Questionnaire 3: 12 months after primary surgery

### Are you currently employed? What applies to you? ***Multiple selection possible, please check for each answer option whether it applies!***

***Please also fill in if you are currently still on sick leave.***

**I am…**

| working fulltime | O |
| --- | --- |
| working parttime | O |
| marginally or irregularly employed | O |
| self-employed/independent | O |
| in a permanent position | O |
| in occupational training, apprentice, or retraining | O |
| in gradual occupational reintegration | O |
| working for another employer (compared to six months ago) | O |
| switched to self-employment | O |
| in a measure offered from the employment agency | O |
| unemployed | O |
| in partial retirement with zero working time | O |
| in pension | O |
| in an internship | O |
| on maternity leave | O |
| other: ____________________________________________________ | O |

### If you have completed a further school education or apprenticeship or your professional position has changed in the last 6 months, please make a note of this:

________________________________________________________________________________________________________________________________________________________________________________________________________________________________________________

### Were you during the past six months on sick leave (counted from now backwards)?

| No | O | I am self employed | O | Yes | O | In total: |  |  | Weeks |  | Days |
| --- | --- | --- | --- | --- | --- | --- | --- | --- | --- | --- | --- |

### Are you currently on sick leave? ***(Please answer all that applies!)***

| No | O |  |
| --- | --- | --- |
| Yes, due to a short-term illness | O |  |
| Yes, because of breast cancer I am not yet back at work | O |  |
| Yes, due to another long (chronic) illness, I have not been at work for months | O |  |
| For another reason, and that is: __________________________________________________________ | | |

### If you have returned to your job or intend to return, what are your reasons for doing so?

### *Non-returnees please tick “Does not apply”* O *Does not apply*

*Please tick an answer in each row!*

| **I return…** | Fully disagree | Agree a little | Moderately agree | Quite agree | | Fully agree | |
| --- | --- | --- | --- | --- | --- | --- | --- |
| because I want to live as I did before the disease | O | O | O | O | O | |  |
| because I am dependent on my earned income | O | O | O | O | O | |  |
| because I enjoy my work | O | O | O | O | O | |  |
| because I need the gainful employment for my pension | O | O | O | O | O | |  |
| because the social environment is important to me | O | O | O | O | O | |  |
| because I feel uncomfortable at home | O | O | O | O | O | |  |
| so that I would have a task | O | O | O | O | O | |  |
| because my daily routine is important to me | O | O | O | O | O | |  |
| Other, and that is: ______________________________________________________________ | | | | | | |  |

### If you are considering not returning to work or have already decided to quit, what are the reasons?

### *Returnees please tick “Does not apply”* O *Does not apply*

*Please tick an answer in each row!*

| **I will (probably) not return…** | Fully disagree | Agree a little | Moderately agree | Quite agree | Fully agree | |
| --- | --- | --- | --- | --- | --- | --- |
| because I lack the physical strength for it | O | O | O | O | O | |
| because I lack the nervous strength for it | O | O | O | O | O | |
| because there are more important things in life | O | O | O | O | O | |
| so that I have more time for my family/friends | O | O | O | O | O | |
| because I do not enjoy work | O | O | O | O | O | |
| because I have no financial need | O | O | O | O | O | |
| I can use my free time reasonably without gainful employment | O | O | O | O | O | |
| I would like to take a break in my professional career | O | O | O | O | O | |
| Other, and that is: _______________________________________________________________ | | | | | |  |

### What treatments have you received within the last six months following your breast cancer surgery? ***(Multiple selections possible!)***

| Further operations on the same  or other breast | O | Other drug treatments | O |
| --- | --- | --- | --- |
| Breast reconstruction | O | alternative practitioners/alternative medicine | O |
| Radiation therapy | O | Rehab/follow-up treatment | O |
| Chemotherapy | O | Self-help group | O |
| Psychotherapy | O | None | O |
| Antihormone therapy | O | Other___________________________ |  |

### Which family status do you have?

| Married and living with my husband | O |
| --- | --- |
| In a registered partnership, living together (same sex) | O |
| Married and living separately from my husband | O |
| Single | O |
| Divorced | O |
| Widowed | O |
| In a registered partnership, not living together (same sex) | O |
| Ended registered partnership (same sex) | O |
| Registered partner died (same sex) | O |

### Are you in a firm relationship?

No O🡪 go to question 50! Yes O Yes, same sex O

### Does your partner live at your house?

No O Yes O

### How many individuals live at your house?

| 1 | 2 | 3 | 4 | 5 | 6 | 7 | 8 | More than 8 |
| --- | --- | --- | --- | --- | --- | --- | --- | --- |
| O | O | O | O | O | O | O | O | O |

### How many individuals living at your house are under 18 years old?

| 0 | 1 | 2 | 3 | 4 | 5 | 6 | 7 | More than 7 |
| --- | --- | --- | --- | --- | --- | --- | --- | --- |
| O | O | O | O | O | O | O | O | O |

### What is the total monthly net income of your household?

### *These include the wages or incomes of all people living and working in the household, as well as rental income, child benefit, parental benefit, unemployment benefit, Hartz IV, social benefit, accommodation costs, housing benefit, pension, etc.*

| under 750 € | 750 to under 1250 € | 1250 to under 1750 € | 1750 to under 2250 € | 2250 to under 3000 € | 3000 to under 4000 € | |
| --- | --- | --- | --- | --- | --- | --- |
| O | O | O | O | O | O | |
| 4000 to under 5000 € | 5000 € and more | I don’t want to answer | I don’t know |  |  | |
| O | O | O | O |  |  |  |

# Questionnaire 4: on average, 5 years after primary surgery

### Are you currently employed? What applies to you? *Multiple choice possible, please check whether each answer option applies!*

***Please also complete this form if you are currently on sick leave as an employed person.***

**I am…**

| Working (employed) | O |
| --- | --- |
| working (independent) | O |
| in gradual occupational reintegration | O |
| in a measure offered from the employment agency | O |
| in pension with paid part-time job | O |
| in Rente without paid part-time job since: Month____ Year______ | O |
| in partial retirement with zero working time | O |
| unemployed | O |
| other:__________________________________________________ | O |

***Continue with question 14***

### Are you currently on sick leave or are you currently unable to work (e.g. as a self-employed person)? *(Multiple selection possible!)*

| No | O | ***Continue with question 15*** |
| --- | --- | --- |
| Yes, due to a short-term illness | O |  |
| Yes, due to breast cancer and continuously since it was diagnosed | O |  |
| Yes, due to the breast cancer, but not all the time, I was back at work in the time in-between | O |  |
| Yes, I have not been at work for months due to another long-term (chronic) illness | O |  |
| Other applies, | O |  |
| and this is: ______________________________________________________ | | |

Even if you are not currently employed, you may have returned to work after your breast cancer diagnosis. If so, when was that?*Returned to work in:*

| Month: |  |  | Year: |  |  |  |  |  | I had not returned to work | O |
| --- | --- | --- | --- | --- | --- | --- | --- | --- | --- | --- |

### Which support services or contact points in your area (within a radius of 25 km) are you aware of and which have you used in the last twelve months?

### *(multiple choice possible!)*

### *Here you will find a list of support services that we assume will help you with everyday impairments.*

*Please put a cross in both sections*

|  | **I am aware of** | | **Have used in the last 12 months** | |
| --- | --- | --- | --- | --- |
|  | yes | No | Yes | no |
| 1. Self-help group | O | O | O | O |

### What treatments have you received within the last six months following your breast cancer surgery? ***(Multiple selections possible!)***

| None | O |  | Bisphosphonate for treating bone resorption | O |
| --- | --- | --- | --- | --- |
| Further operations on the same  or other breast (also breast reconstruction) | O |  | Immune therapy or other specific therapies | O |
| Radiation therapy | O |  | Physiotherapy | O |
| Chemotherapy | O |  | Manual lymphatic drainage | O |
| Psycho-oncological consultation /Psychotherapy | O |  | Rehab/follow-up treatment | O |
| Antihormone therapy, for example *Tamoxifen*, *Letrozol or Anastrozol* | O |  | alternative practitioners/alternative medicine | O |
| Antibodies-Therapy, *for example Herceptin* | O |  | Other:_______________________ | O |

### Which family status do you have?

| Married and living with my husband | O |
| --- | --- |
| In a registered partnership, living together (same sex) | O |
| Married and living separately from my husband | O |
| Single | O |
| Divorced | O |
| Widowed | O |
| In a registered partnership, not living together (same sex) | O |
| Ended registered partnership (same sex) | O |
| Registered partner died (same sex) | O |

### Are you in a firm relationship?

No O🡪 go to question 50! Yes O Yes, same sex O

### Does your partner live at your house?

No O Yes O

### How many individuals live at your house?

| 1 | 2 | 3 | 4 | 5 | 6 | 7 | 8 | More than 8 |
| --- | --- | --- | --- | --- | --- | --- | --- | --- |
| O | O | O | O | O | O | O | O | O |

### How many individuals living at your house are under 18 years old?

| 0 | 1 | 2 | 3 | 4 | 5 | 6 | 7 | More than 7 |
| --- | --- | --- | --- | --- | --- | --- | --- | --- |
| O | O | O | O | O | O | O | O | O |

### What is the total monthly net income of your household?

### *These include the wages or incomes of all people living and working in the household, as well as rental income, child benefit, parental benefit, unemployment benefit, Hartz IV, social benefit, accommodation costs, housing benefit, pension, etc.*

| under 750 € | 750 to under 1250 € | 1250 to under 1750 € | 1750 to under 2250 € | 2250 to under 3000 € | 3000 to under 4000 € | |
| --- | --- | --- | --- | --- | --- | --- |
| O | O | O | O | O | O | |
| 4000 to under 5000 € | 5000 € and more | I don’t want to answer | I don’t know |  |  | |
| O | O | O | O |  |  |  |
