# Supplementary material for: Factors associated with health-related quality of life in women with paid work at breast cancer diagnosis: a German repeated cross-sectional study over the first five years after primary surgery
Source: BMC Cancer. 2025 Jan 17;25:98. doi: 10.1186/s12885-025-13491-8 (PMC11745005; doi:10.1186/s12885-025-13491-8)
Supplement: Supplementary file 2 — Supplementary Material 2 [file 12885_2025_13491_MOESM2_ESM.docx]

**Factors associated with health related quality of life in women with paid work at breast cancer diagnosis: A German repeated cross-sectional study over the first five years after primary surgery**

**Supplement figure 1.** Sensitivity analyses to examine potential survival bias: SF-12 mean summary scores for physical and mental wellbeing at 3 weeks, 6 months, 1 year and 5 years after primary surgery for women who remained at the four time points of the study (Women who were lost to follow up were excluded).

**
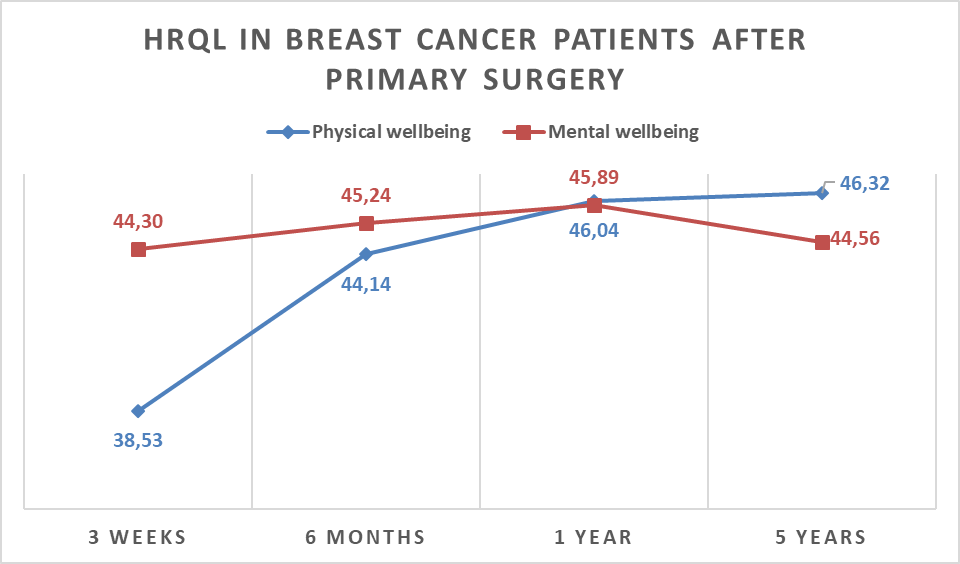
**
